# Prediction value of neutrophil and eosinophil count at risk of COPD exacerbation

**DOI:** 10.1080/07853890.2023.2285924

**Published:** 2023-12-08

**Authors:** Tzu-Tao Chen, Kang-Yun Lee, Jer-Hwa Chang, Chi-Li Chung, Huan Minh Tran, Amja Manullang, Shu-Chuan Ho, Kuan-Yuan Chen, Chien-Hua Tseng, Sheng-Ming Wu, Hsiao-Chi Chuang

**Affiliations:** a Graduate Institute of Clinical Medicine, College of Medicine, Taipei Medical University, Taipei, Taiwan; b Division of Pulmonary Medicine, Department of Internal Medicine, Shuang Ho Hospital, Taipei Medical University, New Taipei City, Taiwan; c Division of Pulmonary Medicine, Department of Internal Medicine, School of Medicine, College of Medicine, Taipei Medical University, Taipei, Taiwan; d School of Respiratory Therapy, College of Medicine, Taipei Medical University, Taipei, Taiwan; e Division of Pulmonary Medicine, Department of Internal Medicine, Wan Fang Hospital, Taipei Medical University, Taipei, Taiwan; f Ph.D. Program in Global Health and Health Security, College of Public Health, Taipei Medical University, Taipei, Taiwan; g Faculty of Public Health, Da Nang University of Medical Technology and Pharmacy, Da Nang, Viet Nam; h International Ph.D. Program in Medicine, College of Medicine, Taipei Medical University, Taipei, Taiwan; i Cell Physiology and Molecular Image Research Center, Wan Fang Hospital, Taipei Medical University, Taipei, Taiwan; j National Heart and Lung Institute, Imperial College London, London, UK

**Keywords:** Acute exacerbation, COPD, eosinophil, inflammation, neutrophil

## Abstract

**Introduction:**

Predicting acute exacerbations (AEs) in chronic obstructive pulmonary disease (COPD) is crucial. This study aimed to identify blood biomarkers for predicting COPD exacerbations by inflammatory phenotypes.

**Materials and methods:**

We analyzed blood cell counts and clinical outcomes in 340 COPD patients aged 20–90 years. Patients were categorized into eosinophilic inflammation (EOCOPD) and non-eosinophilic inflammation (N-EOCOPD) groups. Blood cell counts, eosinophil-to-lymphocyte ratio (ELR), neutrophil-to-lymphocyte ratio (NLR) and neutrophil-to-eosinophil ratio (NER) were calculated. Linear and logistic regression models assessed relationships between health outcomes and blood cell counts.

**Results:**

EOCOPD patients had distinct characteristics compared to N-EOCOPD patients. Increased neutrophil % and decreased lymphocyte % were associated with reduced pulmonary function, worse quality of life and more exacerbations, but they did not show statistical significance after adjusting by age, sex, BMI, smoking status, FEV1% and patient’s medication. Subgroup analysis revealed a 1.372-fold increase in the OR of AE for every 1 unit increase in NLR in EOCOPD patients (*p* < .05). In N-EOCOPD patients, every 1% increase in blood eosinophil decreased the risk of exacerbation by 59.6%.

**Conclusions:**

Our study indicates that distinct white blood cell profiles in COPD patients, with or without eosinophilic inflammation, can help assess the risk of AE in clinical settings.

## 1. Introduction

Chronic obstructive pulmonary disease (COPD), originating from airway inflammation, causes numerous known comorbidities across multiple organs and has been proven systemic in its pathogenesis. A previous study of 304 men with COPD in Spain over five years found that exacerbation frequency played a significant role in mortality, emphasizing the importance of accurately predicting COPD exacerbations [1].

Several studies have been conducted to predict exacerbations. A Korean COPD cohort with 1195 subjects used COPD severity as a multivariate analysis and found that asthma-COPD overlap, moderate COPD and severe COPD were more associated with acute exacerbation (AE) occurrences [2]. A one-year Swiss cohort discovered patients with specific characteristics experienced at least one severe AE but had limitations due to dropout rates [3]. The history of exacerbations remains the single best predictor across all GOLD stages [4]. The ACCEPT model refined by Safari et al., showed better prediction performance than exacerbation history alone [5].

Compared to clinical parameters and pulmonary function test results, blood biomarkers have also been investigated for predicting COPD exacerbation. A study found that serum procalcitonin (PCT) could predict bacterial exacerbation in severe COPD patients [6], while a meta-analysis concluded that CRP and PCT cut-off values were valuable biomarkers [7]. The YKL-40 protein [8] has been suggested as another potential biomarker due to its involvement in bronchial inflammation and remodelling.

In general clinical practice, white blood cell count remains the most accessible option, with elevated eosinophil levels guiding inhaled corticosteroid use according to the latest GOLD report 2023. However, there is limited evidence regarding the predictive value of blood biomarkers for acute COPD exacerbation [9]. Thus, this study aims to analyze multi-centre data from a real-world cohort to investigate the association of different white blood cell counts with COPD exacerbations and identify the most predictive biomarker for exacerbations.

## 2. Material and methods

### 2.1. Ethical considerations

This study was approved by the Taipei Medical University-Joint Institutional Review Board (TMU-JIRB No. N202003075). All participants provided written informed consent, and all procedures were conducted in accordance with the approved protocol.

### 2.2. Study subject

A retrospective case-control study was conducted to examine the association between COPD and certain health conditions. The study included 340 patients with COPD from two hospitals in Taipei between March 2015 and August 2021. Patients had to meet the following criteria were included in this study: (1) confirmation of COPD by a post-bronchodilator forced expiratory volume in the first second (FEV1)/forced vital capacity (FVC) ratio of less than 70% and (2) be between 20 and 90 years of age. Patients with malignancy, bronchiectasis, asthma or any chronic inflammation condition unrelated to COPD (such as chronic renal disease, chronic hepatic disorder, connective tissue disease or immunocompromised/myelosuppressive disorder) were excluded from the study. None of the patients had alpha-1 anti-trypsin deficiency or parasite infection.

### 2.3. COPD outcome measurements

We defined eosinophilic inflammation as a blood eosinophil cut-off value of 2%, as previously reported [10]. The COPD subjects were categorized into two groups: those with eosinophilic inflammation (EOCOPD) and those without eosinophilic inflammation (N-EOCOPD). All COPD patients completed a questionnaire survey conducted by the study coordinator. The survey included measures of quality of life, symptoms and dyspnoea scales, such as the COPD Assessment Test (CAT) [11], the modified Medical Research Council (mMRC) scale [12] and the BODE index (which assesses the body-mass index, degree of airflow obstruction and dyspnoea and exercise capacity) [11]. In addition, we collected their pulmonary function test results, blood cell counts (excluding data within 2 weeks of AE or infection), AE rates (recorded as the number of emergency department visits and hospitalizations for exacerbation management within 1 year), smoking status (recorded as current, ex- or non-smoker) and six-minute walking distance (6MWD) (measured during the six-minute walking test conducted according to the guidelines from the American Thoracic Society [13]). We further calculated the blood eosinophil-to-lymphocyte ratio (ELR), neutrophil-to-lymphocyte ratio (NLR) and neutrophil-to-eosinophil ratio (NER) for each patient.

### 2.4. Statistical analyses

We used a Shapiro–Wilk test to determine the normality of the variables. To minimize the influence of severe outliers, we employed a winsorization approach [14] by replacing extremely low and high values beyond percentiles 1 and 99. Continuous variables that were normally distributed were analyzed using Mann–Whitney *U* test, while categorical variables were compared using the chi-square test with Fisher’s exact approach for post hoc analysis. Linear regression was used to examine the associations of health outcomes with blood cell counts and its ratios (NLR, ELR and NER) in COPD patients. Logistic regression was used to assess the associations of AE with blood cell counts and its ratios (NLR, ELR and NER) in COPD patients or EOCOPD and N-EOCOPD patients. The models were adjusted for co-variables including age, sex, BMI and smoking status. All statistical analyses were performed using SPSS version 26 for Windows statistical software (SPSS Inc., Chicago, IL, USA). A *p* value less than .05 was considered statistically significant.

## 3. Results

### 3.1. Characteristics of study subjects

The characteristics of all 340 patients enrolled in the study are shown in Table 1.Of these, 185 patients had COPD with eosinophilic inflammation and 155 patients had COPD with non-eosinophilic inflammation. The average age of these patients was 71.7 years, and 88.24% were male. The mean BMI was 23.53 kg/m^2^, with 32.6% being current smokers, 56.5% ex-smokers and 10.9% non-smokers. In pulmonary function tests, the mean FEV_1_ was 61.73%, mean FVC was 84.11% and mean FEV_1_/FVC was 56.91%. For COPD outcome measurement, the mean 6MWD, mMRC, CAT and BODE index were 403.38 m, 1.44 points, 11.07 points and 4.66 points, respectively. The average AE of COPD was 0.55 times/year. In blood white cell counting, the average neutrophils, eosinophils, lymphocytes, ELR, NLR and NER were 65.45%, 2.81%, 22.27%, 0.13, 5.05 and 123.8, respectively. Compared to the COPD patients with non-eosinophilic inflammation, the patients in the eosinophilic inflammation group were younger, had a longer 6MWD, lower mMRC score, lower WBC counts, lower neutrophil %, higher eosinophil % and lymphocyte %, higher ELR, lower NLR, lower NER and less oral corticosteroids in prescription (all *p* < .05).

**Table 1.** Characteristics of COPD patients.

Variables	Total COPD patients (*n* = 340)	Eosinophilic inflammation in COPD (*n* = 185)	Non-eosinophilic inflammation in COPD (*n* = 155)	*p* Value
Age, years	71.7 ± 10.4	70.6 ± 9.9	73 ± 10.7	*p* < .05
Male, *n* (%)	300 (88.24)	168 (90.81)	132 (85.16)	.129
BMI, kg/m^2^	23.53 ± 4.17	23.76 ± 4.33	23.25 ± 3.95	.396
*Smoking status, n (%)*				.234
Current smoker	111 (32.6)	59 (31.9)	52 (33.5)	
Ex-smoker	192 (56.5)	101 (54.6)	91 (58.7)	
Non-smoker	37 (10.9)	25 (13.5)	12 (7.8)	
*Lung function*				
FEV_1_, %	61.73 ± 19.87	62.61 ± 19.9	60.68 ± 19.78	.330
FVC, %	84.11 ± 21.33	85.49 ± 21.12	82.46 ± 21.47	.210
FEV_1_/FVC, %	56.91 ± 12.98	56.87 ± 13.22	56.97 ± 12.68	.966
6MWD, m	403.38 ± 116.15	428.16 ± 111.81	373.98 ± 114.33	*p* < .05
mMRC, point	1.44 ± 1.06	1.34 ± 1.11	1.56 ± 0.98	*p* < .05
CAT, point	11.0 ± 7.46	11.11 ± 7.77	10.88 ± 7.08	.988
BODE index, point	4.66 ± 2.38	4.45 ± 2.49	4.90 ± 2.20	.075
AE, *n*	0.55 ± 1.58	0.48 ± 1.38	0.65 ± 1.78	.682
WBC, /mm^3^	8195 ± 3156	7254 ± 2076	9318 ± 3793	*p* < .05
Neutrophil, %	65.45 ± 12.59	59.87 ± 9.95	72.07 ± 12.16	*p* < .05
Eosinophil, %	2.81 ± 2.36	4.44 ± 2.01	0.87 ± 0.6	*p* < .05
Lymphocyte, %	22.27 ± 10.22	25.72 ± 9.0	18.15 ± 10.07	*p* < .05
ELR	0.13 ± 0.13	0.2 ± 0.14	0.05 ± 0.05	*p* < .05
NLR	5.05 ± 11.16	2.85 ± 1.75	7.67 ± 16.03	*p* < .05
NER	123.8 ± 250.9	16.8 ± 8.38	251.5 ± 328.7	*p* < .05
*Medication*				
OCS, *n* (%)	33 (9.7)	12 (6.5)	21 (13.5)	*p* < .05
ICS, *n* (%)	216 (63.5)	126 (68.1)	90 (58.1)	.07
LABA, *n* (%)	290 (85.3)	160 (86.5)	130 (83.9)	.540
LAMA, *n* (%)	263 (77.4)	138 (74.6)	125 (80.6)	.196

Abbreviations: BMI, body-mass index; FEV_1_, forced expiratory volume in 1 s; FVC, forced vital capacity; 6MWD, six-minute walk distance; mMRC, modified medical research council; CAT, COPD assessment test; BODE, body-mass index, airflow obstruction, dyspnoea and exercise; AE, acute exacerbation; WBC, white blood cell; ELR, eosinophil/lymphocyte ratio; NLR, neutrophil/lymphocyte ratio; NER, neutrophil-to-eosinophil ratio; OCS, oral corticosteroid; ICS, inhaled corticosteroid; LABA, long acting beta agonist; LAMA, long acting muscarinic antagonist.

### 3.2. Associations of blood cells counts and COPD outcomes

In Table 2, we present the results of our analysis on the associations between white blood cell counts and various clinical parameters of COPD. We found that a 1% increase in neutrophil count was associated with a significant decrease in lung function, as evidenced by a 0.219% decrease in FEV_1_ (95% CI: −0.511, −0.181, *p* < .05), a 0.163% decrease in FVC (95% CI: −0.456, −0.098, *p* < .05) and a 0.164% decrease in post-bronchodilator FEV_1_/FVC (95% CI: −0.278, −0.060, *p* < .05). Additionally, a 1% increase in neutrophil count was associated with a worse symptom burden, with a 0.236 m (95% CI: −3.568, −0.571, *p* < .05) decrease in 6-min walking distance, and a 0.273 point increase in mMRC score (95% CI: 0.014, 0.032, *p* < .05) and a 0.194 point increase in CAT score (95% CI: 0.052, 0.179, *p* < .05). Neutrophil count was also positively associated with the BODE index (0.232 points increase, 95% CI: 0.024, 0.063, *p* < .05) and the frequency of AE (0.119 times increase, 95% CI: 0.002, 0.028, *p* < .05). On the other hand, we found that a 1% increase in eosinophil count was associated with a significant improvement in exercise capacity, with a 0.293 m increase in 6MWD (95% CI: 6.020, 22.169, *p* < .05). A higher eosinophil count was also associated with a lower symptom burden, as evidenced by a 0.135 point decrease in mMRC score (95% CI: −0.108, −0.012, *p* < .05). Finally, we found that a 1% increase in lymphocyte count was associated with a significant improvement in lung function, as evidenced by a 0.237% increase in FEV1 (95% CI: 0.258, 0.662, *p* < .05), a 0.183% increase in FVC (95% CI: 0.162, 0.601, *p* < .05) and a 0.168% increase in post-bronchodilator FEV1/FVC (95% CI: 0.080, 0.347, *p* < .05). A higher lymphocyte count was also associated with a lower symptom burden, as evidenced by a 0.257 point decrease in mMRC score (95% CI: −0.038, −0.016, *p* < .05) and a 0.180 point decrease in CAT score (95% CI: −0.211, −0.054, *p* < .05). Additionally, lymphocyte count was negatively associated with the BODE index (0.237 points decrease, 95% CI: −0.079, −0.031, *p* < .05) and the frequency of AE (0.147 times decrease, 95% CI: −0.039, −0.006, *p* < .05). One unit increase in ELR was associated with a 0.192 m longer distance of 6MWD (95% CI: 15.255, 285.155, *p* < .05).

**Table 2.** Associations between white blood cells counts and lung function, six-minute walk distance, CAT and BODE index.

	Neutrophil *β* Coefficient (95% CI))	Eosinophil *β* Coefficient (95% CI))	Lymphocyte *β* Coefficient (95% CI)	ELR *β* Coefficient (95% CI)	NLR *β* Coefficient (95% CI)	NER *β* Coefficient (95% CI)
*Lung function*						
FEV_1_, %	−0.219 (−0.511, −0.181)*	0.087 (−0.164, 1.633)	0.237 (0.258, 0.662)*	0.009 (−14.881, 17.664)	−0.057 (−0.292, 0.088)	−0.072 (−0.014, 0.003)
FVC, %	−0.163 (−0.456, −0.098)*	0.096 (−0.096, 1.832)	0.183 (0.162, 0.601)*	0.043 (−10.367, 24.538)	−0.063 (−0.325, 0.083)	0.061 (−0.014, 0.004)
FEV_1_/FVC, %	−0.164 (−0.278, −0.060)*	0.015 (−0.504, 0.674)	0.168 (0.080, 0.347)*	−0.049 (−15.438, 5.790)	−0.035 (−0.165, 0.084)	−0.046 (−0.008, 0.003)
6MWD, m	−0.236 (−3.568, −0.571)*	0.293 (6.020, 22.169)*	0.231 (0.691, 4.652)*	0.192 (15.255, 285.155)*	−0.078 (−1.721, 0.661)	−0.147 (−0.125, 0.011)
mMRC, point	0.273 (0.014, 0.032)*	−0.135 (−0.108, −0.012)*	−0.257 (−0.038, −0.016)*	−0.022 (−1.052, 0.693)	0.062 (−0.004, 0.016)	0.100 (0.000, 0.001)
CAT, point	0.194 (0.052, 0.179)*	−0.052 (−0.506, 0.176)	−0.180 (−0.211, −0.054)*	0.008 (−5.693, 6.595)	0.059 (−0.033, 0.111)	0.043 (−0.002, 0.005)
BODE index, point	0.232 (0.024, 0.063)*	−0.091 (−0.199, 0.016)	−0.237 (−0.079, −0.031)*	0.016 (−1.654, 2.236)	0.033 (−0.016, 0.030)	0.035 (−0.001, 0.001)
AE, *n*/yr	0.119 (0.002, 0.028)*	−0.045 (−0.102, 0.041)	−0.147 (−0.039, −0.006)*	0.068 (−0.467, 2.111)	0.025 (−0.012, 0.019)	0.040 (0.000, 0.001)

Abbreviations: FEV_1_, forced expiratory volume in 1 s; FVC, forced vital capacity; 6MWD, six-minute walk distance; mMRC, modified medical research council; CAT, COPD assessment test; BODE, body-mass index, airflow obstruction, dyspnoea and exercise; AE, acute exacerbation. Linear regression adjusted with age, sex, BMI, FEV1%, medication and smoking status.

* *p* Value <.05.

### 3.3. Associations of AE with blood cell counts in COPD

In Table 3, we analyzed the OR of AE in people with COPD based on their blood white cell levels. Our analysis found that a 1% increase in neutrophil levels was associated with a 0.029 times increase in the crude OR of AE (95% CI: 1.008, 1.050, *p* < .05), while a 1% increase in lymphocyte levels was associated with a 0.037 times decrease in the crude OR of AE (95% CI: 0.937, 0.989, *p* < .05). But after adjusting for age, sex, BMI, FEV1%, use of OCS, ICS, LABA, LAMA and smoking status, we found no statistically significance.

**Table 3.** Odds ratio of acute exacerbation of COPD with differential white blood cell counts.

	Crude OR (95% CI)	Adjusted OR (95% CI)
Neutrophil	1.029 (1.008, 1.050)*	1.022 (0.998, 1.046)
Eosinophil	0.932 (0.832, 1.044)	0.945 (0.838, 1.066)
Lymphocyte	0.963 (0.937, 0.989)*	0.970 (0.941, 1.000)
ELR	2.204 (0.357, 13.595)	2.889 (0.382, 21.830)
NLR	1.004 (0.984, 1.025)	1.004 (0.981, 1.028)
NER	1.001 (1.000, 1.002)	1.001 (1.000, 1.002)

Abbreviations: ELR, eosinophil/lymphocyte ratio; NLR, neutrophil/lymphocyte ratio; NER, neutrophil-to-eosinophil ratio. Logistic regression adjusted with age, sex, BMI, FEV1%, medication and smoking status.

* *p* Value <.05.

### 3.4. Associations of AE with blood cell counts in EOCOPD and N-EOCOPD

Table 4 presents the OR of AE in the EOCOPD and N-EOCOPD groups. Our analysis found that after adjusting for age, sex, BMI, FEV1%, use of OCS, ICS, LABA, LAMA and smoking status, in the EOCOPD group, a 1 unit increase in the NLR increased the OR of AE by 1.372-fold (95% CI: 1.092, 1.618, *p* < .05); where in the N-EOCOPD group, a 1% increase in blood eosinophil decreased the risk of AE by 59.5% (OR 95% CI: 0.180, 0.904, *p* < .05), and a 1 unit increase in NER increased the risk of AE by 0.1% (OR 95% CI: 1.000, 1.003, *p* < .05).

**Table 4.** Odds ratio of acute exacerbation of COPD with differential blood cell counts in the EOCOPD or N-EOCOPD groups.

	EOCOPD		N-EOCOPD	
	Crude OR (95% CI) Adjusted OR (95% CI)		Crude OR (95% CI) Adjusted OR (95% CI)	
Neutrophil	1.03 (0.993, 1.068)	1.027 (0.985, 1.070)	1.044 (1.009, 1.080)*	1.040 (0.998, 1.084)
Eosinophil	0.919 (0.768, 1.099)	0.865 (0.709, 1.055)	0.422 (0.218, 0.817)*	0.404 (0.180, 0.904)*
Lymphocyte	0.958 (0.919, 1.00)*	0.956 (0.910, 1.003)	0.956 (0.917, 0.996)*	0.967 (0.919, 1.018)
ELR	5.944 (0.623, 56.706)	6.025 (0.502, 72.361)	0.002 (0.000, 39.472)	0.000 (0, 000, 17.265)
NLR	1.309 (1.085, 1.579)*	1.372 (1.102, 1.709)*	1.000 (0.978, 1.024)	1.001 (0.973, 1.029)
NER	1.022 (0.981, 1.064)	1.026 (0.982, 1.072)	1.001 (1.000, 1.002)*	1.002 (1.000, 1.003)*

Abbreviations: ELR, eosinophil/lymphocyte ratio; NLR, neutrophil/lymphocyte ratio; NER, neutrophil-to-eosinophil ratio. Logistic regression adjusted with age, sex, BMI, FEV1%, medication and smoking status.

* *p* Value <.05.

## 4. Discussion

In this study, we examined the association of different white blood cell counts with COPD outcomes. The significance of this study is that we observed that N-EOCOPD, with increasing NER and decreasing eosinophil counts, had a higher risk for AE, whereas EOCOPD, with increasing NLR, was associated with increasing AE. The distinct white blood cell profiling in COPD, with or without eosinophil inflammation, could be used to assess the risk of AE in clinical settings.

In this cross-sectional study, we aimed to investigate the associations between different white blood cell counts and various COPD outcomes. Our results showed that increased peripheral neutrophil count was linked to changes in pulmonary function and worse quality of life, as measured by mMRC, CAT and BODE index, as well as an increased risk of AE in COPD patients. These findings are consistent with previous research indicating that elevated neutrophil counts were associated with higher exacerbation and mortality rates in COPD [15,16]. Additionally, our study is consistent with previous research demonstrating that increased peripheral neutrophil count is associated with decreased pulmonary function in normal adult populations [17] and asymptomatic smoking adults [18].

Interestingly, our study also revealed that higher peripheral eosinophil counts were associated with a longer 6MWD and better quality of life, as measured by mMRC and BODE index, which is consistent with a prior study that reported better improvement of 6MWD after rehabilitation in COPD patients with higher eosinophil counts [19]. This finding contrasts with a prior study that reported higher St George Respiratory Questionnaire symptom scores in COPD patients with higher eosinophil counts [20]. In an earlier study, Semenzato et al. used 1800 cells/µL as the blood lymphocyte cut-off and discovered that cigarette smokers with COPD had lower lymphocyte counts than healthy smokers, and the decline of lymphocytes was greater in the COPD group, which was also related to a higher prevalence of cancer and mortality [21]. We further found that higher peripheral lymphocyte counts were associated with increased pulmonary function test, better quality of life (by mMRC, CAT, BODE index and 6MWD), and decreased AE.

Previous studies have addressed the use of NLR as a marker for variable COPD outcomes. A recent meta-analysis reported that NLR was associated with an increased risk of mortality and exacerbation [22]. On the other hand, due to the high variability of blood eosinophil, some researchers suggested that ELR, eosinophil/monocyte ratio (EMR) and ENR in blood were better indicators for defining the eosinophilic subtype in COPD exacerbations [23]. However, in our study, we did not find a significant correlation of NLR, ELR or ENR with AE of COPD. The results suggest that different white blood cell counts depend on the phenotypes and progression of COPD.

Next, we observed that COPD patients with higher eosinophil and lower lymphocyte counts had a crude higher risk for AE, but became statistically insignificance after adjusted with age, sex, BMI, FEV1%, medication and smoking status, suggesting there might be some confounders to interfere the different expression. To investigate this further, we conducted a subgroup analysis based on eosinophil inflammation in COPD. Interestingly, we found that a decrease in eosinophil was associated with an increased risk of AE in the N-EOCOPD group but not in the EOCOPD group. This is different from a prior study [24] that found a positive correlation between baseline eosinophil count ≥300/ul in stable disease and eosinophilic exacerbation in the EOCOPD group. These results highlight the importance of considering eosinophilic inflammation in COPD subtyping to better understand the association between white blood cell counts and AE risk.

Our study found that an increase in NLR was a significant indicator of AE in the EOCOPD but not in the N-EOCOPD group. This is in contrast to a prior study [25], which reported the value of NLR as an indicator of exacerbation severity in neutrophilic COPD group (defined as blood eosinophil <2%). The NLR cut-off value in their study was defined as 8.01, which had 78% sensitivity and 60% specificity for in-hospital mortality in COPD exacerbation. However, the discrepancy between Aksoy’s study and ours could be due to the proportion of active smokers, as NLR was highly correlated with systemic inflammation in active smokers [26]. In our study, the inclusion criteria comprised only 32.8% current smokers, which had a minimal impact on selection bias. These findings suggest that NLR could be used as an indicator of AE severity in EOCOPD but not in N-EOCOPD, and that smoking status should be considered when using NLR as a biomarker in COPD.

A notable limitation of our study is the inclusion of patients who were using oral and inhaled corticosteroids without accounting for the daily dosage. Specifically, the use of oral corticosteroids was more prevalent in the N-EOCOPD group (13.5% compared to 6.5% in the EOCOPD group), likely due to a higher rate of exacerbations in these patients. This is evidenced by the average annual exacerbation rates: 3 versus 0.28 in the N-EOCOPD group and 2.6 versus 0.3 in the EOCOPD group, with all differences being statistically significant (*p* < .05). Another limitation was the absence of serum IgE measurements, which prevented us from determining the atopy proportion in our study population. Although we excluded patients with a history of asthma, some may still have had underlying airway hyperresponsiveness. Additionally, the study was limited by a small sample size, suggesting the need for larger-scale research in the future. Lastly, we did not investigate the prevalence of pneumonia, a known complication of corticosteroid use that can contribute to increased exacerbations in COPD. Further research is required to explore the interplay between these biomarkers and the exacerbation mechanisms in COPD-related inflammation.

## 5. Conclusion

In conclusion, our study suggests that distinct white blood cell profiling in COPD, with or without eosinophil inflammation, can be used to assess the risk of AE in clinical settings. In N-EOCOPD patients, low eosinophil and high NER in peripheral blood, and in EOCOPD patients, high NLR were valuable predictors for COPD exacerbations.

## Ethics approval and consent to participate

This study protocol was approved by the Taipei Medical University-Joint Institutional Review Board.

## Data Availability

Data available on request from the authors.
